# Medical Jurisprudence

**Published:** 1907-06

**Authors:** E. S. McKee

**Affiliations:** Cincinnati


### PROGRESS IN MEDICAL SCIENCE.

### Medical Jurisprudence.

BY E. S. MCKEE, M.D., Cincinnati.

[*Lancet-Clinic.*]

THE MEDICAL EXPERT AND HIS TRIALS.

L. B. Sawyer, M.D., Professor of Medical Jurisprudence in the University Medical College, Kansas City, Mo., discusses this subject. He says much has been written in the medical world of the abuses by what is known as the professional witness or expert. Physicians are not, as a rule, untruthful; indeed, my experience has been that they are of as high a standard as men in any walk of life, but many of them seem to have a peculiar conception of their duty when testifying in court. Unfortunately, the rule is that they take one side or the other of each case, and in their desire to convince the court and jury of their views, go to great lengths in evading direct answers, and even greater in evading the direct issue. A physician recently said to me: "Expert testimony is a farce from any standpoint." A board of managers has been suggested to whom all legal disputes, in which a medical question is involved, should be referred.

Other questions, fully as impracticable have been suggested, but none has yet been offered that seems to meet the situation. In every community where medical testimony is used to any great extent there are well-established and active medical societies. Let each such society have a standing committee on medical testimony. Let this committee furnish the most recent evidence obtainable to the association. It need not be used with a view of exposing the witness to ridicule, nor for criticism, but for the purpose of educating the profession on the questions that are in litigation. The result will be that physicians will be brought to know that the rash statements which they make to innocent (?) lawyers may be discussed by their brothers, and such statements as given by a prominent physician on the witness stand will not be made. Who will deny that publicity will not check such practices, as it has done in politics and graft and trusts?

---

MEDICAL EXPERT EVIDENCE AND THE BILL PENDING BEFORE THE MAINE LEGISLATURE.

THIS is the subject of an address before the Fellows of the Maine Academy of Medicine and Science by the Hon. Clark Bell of New York, president of the New York Medico-Legal Society and editor of the New York *Medico-Legal Journal.* He enumerates the following to which he objects:

1. Partisanship of medical experts.

2. The payment of large sums to medical expert witnesses for their services.

3. The growing practice of having physicians attend constantly while evidence is being taken and then asking them what is their professional opinion upon all the evidence thus heard by them.

4. The hypothetical question.

The value and efficiency and public utility of medical expert testimony has fallen in great disfavor among all classes of our people in a lamentable degree, a fact which no member of the profession of law or medicine can longer disregard or ignore. So long as medical experts can be found who will swear directly opposite to each other, it is time for legislation to arrest the evil. The bill before the legislature of the state of Maine, which is under discussion in the paper by Mr. Bell, provides for the appointment of one or more examiners in cases where an expert opinion or evidence is admissible. The examiner, at the request of either party or the court appointing him, shall make such examination or study of the subject matter or question as he deems necessary for a full understanding thereof, and such further reasonable pertinent examination as either party shall request. Reasonable notice shall be given each party of physical examination of persons, things and places, and each party may be represented at such places. The bill provides that these examiners shall be paid by the state and the amount of their compensation fixed by the judge who tries the case. It is noticed that the Maine bill does not prevent the employment of other expert testimony outside the triers named by the court, if either party so desires, but it is perfectly plain that very little attention would be paid by the jury to experts who were paid large sums to influence their opinions if they run contrary to the opinions of the triers appointed by the court. Of what good, then, would be the millionaire's millions?

---

### WHY IS THE MEDICAL EXPERT DISCREDITED IN COURT?

This is the title of a paper by W. R. Allen, Judge of the Superior Court of North Carolina. While it is true that the medical expert is discredited at court, it is not the law that discredits him. It says distinctly that in speaking of matters within the domain of his profession and knowledge that his evidence should have peculiar weight with the jury. It is when speaking of matters not within his own knowledge and when he expresses an opinion based upon the evidence of others that he suffers most at the hands of the judge and jury, although he is not always safe when expressing an opinion based upon his own knowledge. Owing to the rapid growth of the knowledge of medicine, what is true today may not be true tomorrow, and thus the medical expert must make his statements with doubts and qualifications which add doubt to the minds of the jury. It is generally from the ignorant or unscrupulous that you may get the positive opinion. I am inclined to believe that the hypothetical question ought to be entirely abolished.

A judge and jury are not inclined, in worldly matters, to have much faith in those things they do not understand. The expert stands discredited unless he is understood. The remedy for the expert is simplicity of language and clearness of expression. An expert's opinion may be entirely theoretical, based on reading alone, and, strange to say, the books in his profession on which he relies may not be introduced to contradict him. The expert should be able to show practice and experience as well as theory. The remedy for this is in the medical profession, as they alone have the ability to know who among them are real experts. The medical expert is, strange to say, made judge of his own competency. The expert should be called by the court to be a witness.

---

### THE SOLUTION OF THE MEDICAL EXPERT.

This question is still *sub judice.* He is tolerated and frequently dismissed without honor. The court should select experts to advise with him and the jury should be instructed in this same way. If the expert is judicious and skilful and has judgment and wisdom, his services will be valued highly and ought to be paid in the same manner. If he is incompetent the court will not be long in discovering the fact. Ridicule will drive the incompetent, dishonest, one-sided witness from the court-room unless something is done soon to improve his standing. A few experiences like the one in the early part of the Thaw case will either elevate the expert witness or drive him into oblivion. The mistake on the part of the first unfortunate expert in the Thaw case was to allow himself to be drawn into the case so early. He should have refused to testify on the sanity of the defendant until he had heard all the testimony upon which he should base his opinion. No medical man should go on the stand to testify as an expert unless he is prepared to defend his opinion by his own personal experience, knowledge and observation.

An expert witness, if he expects to instruct the court and jury, should be educated and qualified at least in the specialty he represents. If incompetent and unworthy of his position, he should expect to be handled unmercifully. It is generally impossible for attorneys to understand a medical situation and to comprehend the necessity of framing questions which are rational, and calculated to bring out the point at issue. The attorneys answer that they are afraid of bringing out too much for fear that the opposition may profit, and thus the expert is handicapped, and the court and jury are so confused that they are glad to eliminate the expert testimony and decide for themselves. An expert witness may be able and competent but easily embarrassed, and therefore at the mercy of the attorney. The court, if appealed to, will always protect a witness from an unjust lawyer. The expert who is anxious to air his opinions and show his wisdom is always in danger of cross-fire and confusion. The stand an expert must take is for honesty and sincerity of purpose, a fairness to give the required information in both direct and crossexamination, and to be plain and concise in his statements and to be able to show that his opinion is backed by knowledge.

---

### THE ATTITUDE OF THE MEDICAL EXPERT WITNESS TOWARDS COURT AND COUNSEL.

THERE have been reported instances where a medical man by his superior wit has brought confusion upon an impudent lawyer, but usually the trial of a lawsuit is a serious business, and it is better for the witness to take the matter seriously. In case the attorney in his examination exceeds what the witness thinks are reasonable bounds, the latter may control the situation by refusing to say anything till he has appealed to the court for protection. Most all judges are quick to protect a witness who has been unfairly dealt with. There is a great deal of nonsense about a witness being mixed up by a lawyer. An unsatisfactory exhibition that a witness may make on the stand is usually due to his own ignorance or dishonesty. If he knows his subject and is trying to testify honestly, it is difficult to confuse a witness of ordinary coolness. If the medical witness observe these suggestions it will go far toward remedying the evils at present connected with expert testimony and to preserve, untarnished, the reputation of the physician himself.

---

### EXPERT EVIDENCE.

THIS trite subject is receiving unusual attention of late. That great cosmopolitan bad smell, the Thaw trial, has renewed comment on this subject. Dr. Wiley who was first to take the stand, expresses his position best by his own plaintive plea during the grilling he received: "I came here as a witness of fact, and they have made me an expert without any preparation." The *Buffalo Medical Journal says*: "Dr. Wiley as a witness was an error; as an expert he was a gelatinous failure." Dr. Evans has invented the terms "brain storm" and "mental explosion," expressions which will probably stay with us and become additions to our rich vocabulary of slang. "Wileyism" is a term which has come to stay, and means something which is purely asinine.

---

### EXPERT WITNESSES AND FEES.

IN Toronto, according to the *Canadian Journal of Medicine and Surgery,* we have in the case of a recent telephone girls' strike a quota of medical practitioners summoned by the counsel on each side to give testimony on such points as to whether eight hours were too much for the average operator to work. Of course, diverse opinions were readily obtained. The counsel for the girls took the ground that the physicians summoned were entitled to collect only the usual $4.00 per diem allowed medical witnesses in Canada, and not an expert fee. This brings up the old question: What is a physician entitled to for attendance in court? This depends largely upon the character of the evidence he is required to give. Four dollars per day is surely insufficient. What would be the average lawyer's fee for such service? Allowing that this is a fair fee, it is entirely different if the medical man is to give expert testimony. An ordinary witness must only state facts; but when he is called upon to give an opinion as to matter depending on special knowledge he has a right to be paid accordingly. Courts have ruled very differently on the subject of expert fees. Some have ruled that the expert is not only a compellable witness, but can only claim the ordinary fees. Judge Morson, of Toronto, on the other hand, gave judgment for two Toronto physicians who sued for expert fees of $50.00 each per day, giving it as his opinion that an expert, if an expert, was entitled to collect a reasonable sum for his fee.

Let the profession stand up and fight for some reasonable remuneration, say five dollars per hour, and all stand together, and we will get it. Alfred Swayne Taylor once said that no man had a right to enter the expert witness-box unless he knew all about the subject under discussion. That is too sweeping a definition of an expert.

---

### EXPERT MEDICAL TESTIMONY.

This was the subject of the presidential address before the Medical Association of Central New York, by Dr. David M. Totman. The doctor said that he had been advised by one of his friends, a most learned member of the bar, that there was neither honor, glory nor anything else to be gotten out of a medico-legal case, but to get one's name smirched and dishonored. The conditions which surround the giving of expert testimony in our courts are well-nigh intolerable, considering the attitude of the public press, the insulting innuendos of graft, and the charges of open perjury committed by medical men on the stand. He saw no relief under the present system of jury trial. It will take centuries of education and evolution for the average jury to understand much expert testimony, so, no difference how perfect the expert testimony given the jury, the verdict will not be influenced one whit. Even the learned judges are not always able to appreciate the bearings of expert medical testimony. He holds that hysteria is a subject of such a nature that neither judge nor jury, however learned, may be able to execute justice when such a subject is under consideration. No man can pass upon the value of expert testimony in its entirety unless he has had fifteen or twenty years of study, observation and management of hysteria. The appellate courts should be given the power by legislation to name a board of three members for each appellate division of properly qualified physicians of fifteen or twenty years' experience, whose proper office and duty would be to consider all medico-legal cases, under the jurisdiction of the appellate court, as advisors to the court. In some way there should be established a proper review on the medico-legal evidence, which he is free to say does not obtain at the present time. This would be a death blow to ambulance chasers. Shrewd lawyers have profited for years from the fact that medico-legal evidence has been measured by bulk and not by quality.

Such examples as one of the experts in the Thaw trial redound greatly to the discredit of the medical profession. The profession is filled with thoroughly competent men who could render good account of themselves as experts in whatsoever line they may be engaged, and it is very unfortunate that in a case so conspicuous as that of the present instance the mistake should have been made of bringing to the witness-stand one so capable of holding the profession up to ridicule. The random selection of medical experts should be discontinued. Inquiry among the practitioners of one's own city will generally give an insight into one's real capacity. How often have we seen mistakes made in the selection of medical experts as well as the selection of physicians of consultants by the laity! No one knows a medical man's capacity so well as his colleagues.

---

### NON-PARTISAN EXPERTS.

Dr. Haldor Sneve, of St. Paul, in the *St. Paul Medical Journal* for April, 1907, reports the following case of interest:

A woman was caught by a guy wire which had been built by the patient's brother and other relatives and sold to a large telephone corporation. Two non-partisan lady physicians were appointed by the court to examine the patient. They testified that they found no physical signs whatever of adhesions or any trouble with the lungs or any difficulty discoverable in the back part of the head, both of which the patient claimed. They said the patient complained of pain on pressure over the occiput and under the right clavicle. The jury awarded $2,000 damages. In other words, in spite of the negative testimony of two non-partisan physicians, the jury awarded damages too large to be merely punitive for having a guy wire on the public highway. He found the judge's action a step in the right direction, but thinks that if a jury of doctors appointed by the court could examine the patient, listen to the testimony and then testify without having any other medical testimony, that would come nearer the goal. The writer thought that all questions as to amount and character of physical injury should be passed on by a medical and not a lay jury. The jury should pass on the facts of the case, and the judge, after the testimony of the medical jury, should award the damage, if any.

---

### SUGGESTIONS TO THE MEDICAL WITNESS.

This is the subject of a paper by Surgeon-Major Wrafter (retired) in *Practical Medicine*, Delhi, India. An expert witness should bear in mind that he is not assisting the administration of justice if he presents a partial statement of the facts, and he should be conservative in his statements. One's confidence in the medical profession is impaired when he sees medical men go on the stand and give positive and decisive answers to questions which he knows can only be answered doubtfully. The medical witness frequently has an overpowering desire to know all. The direct answer, "I don't know," is often the best evidence of the superior intelligence of the medical witness. In the sickroom the physician is the ultimate authority, and it is sometimes better that he should make a mistake than that he should vacillate or waver. On the witness-stand the situation is different. If the witness, before undertaking to give an answer, would insist upon understanding the question thoroughly he would in many cases, if he gave his honest deliberate opinion, answer that the question was absurd and he could not answer it. If this is true, that is just the kind of an answer which should be given. Furthermore, it is practically impossible for the medical man who knows anything about the case in hand to eliminate from his consideration, when he answers a hypothetical question, matters which are not included in the question, yet the law presumes that, in answering a hypothetical question, the witness is assuming only the facts that are stated in the question. This tendency to answer a different question from the one put to the witness is a fruitful source of controversy.

---

### A FEW REMARKS ON MEDICAL EXPERT TESTIMONY.

George Franklin Shiels (*Medical Record*) considers this subject under various headings. He believes that expert medical evidence could be of the greatest value in upholding justice if it were properly introduced. He suggests two plans. (1). Let the attorneys of each side select two experts, and let the four thus chosen choose a fifth. These five men could, after mature deliberation, present a full and useful report on any technical points considered by them; or (2) the matter could be left in the hands of the court, who could call one or a dozen medical men to elucidate without prejudice any technical points that might arise in the trial. The writer favors the plan in which the court has control.

---

### BOARDS OF EXPERTS.

Judge Allen, in his paper, gives "into the hands of the medical profession the power of naming the board of experts to the judge," while Dr. Totman would place this power entirely in the hands of the judge himself. Judge Allen was, indeed, quite emphatic on this point, claiming that the profession knew the standing of its members and the capability of each for a place on such a board, and as the profession was, as a whole, to a large extent interested in expert testimony, it ought to say whom it will have to represent it. Both suggestions are from eminent authorities, one from the side of the law and the other from that of medicine, and both have some strong points to recommend them. Either would be acceptable if it would remove the odium now attached to expert testimony before the courts.

There is a movement on foot in Kentucky outlining steps to be taken that whenever there is a question up in court where the truth can only be arrived at as the result of a competitive medical opinion the latter question would be referred to a board of three or five men from the medical profession, selected by the presiding judge, these men not to be approached by the lawyers on either side or allowed to testify in court at all. Let these men have the facts all placed before them; let them examine the prisoner or plaintiff as the case may be, and let them submit a signed and sworn-to statement of their findings and opinions which should be read to the jury; the compensation of the medical board to be included in the costs of the case.

---

### THE HYPOTHETICAL QUESTION.

THE literally lettered editor of the *St. Louis Medical Review* discusses the above subject thus ably:

"It is time that some limitation was placed to that double distilled essence of tomfoolery, the so-called 'hypothetical question' put to expert witnesses in courts of law. Its ostensible object is to lay before the witness in abstract form certain conditions supposed to have existed in the concrete case before the court, so that the judgment of the expert may be obtained on the material facts requiring expert opinion involved, released from all complicating extraneous circumstances that might confuse the issue or call bias into play. Its practical effect is more often to obscure the main scientific issue by the cuttle-fish expedient of 'ink slinging,' in the production of mases of verbosity that by comparison reduced the involved sentences of Livy to a laconic staccato. When a certain counsel in a present *cause celebre* is allowed, as the press reports state, to occupy one hour and eighteen minutes in reading one such 'hypothetical question,' the details of which it is impossible for any man living to carry in his head, it is safe to assert that the elucidation of truth is the last thing he had in view. No hypothetical question should be allowed to be of greater length than a majority of the jury, being ordinarily intelligent persons, could repeat by heart after having it two or three times read to them. If the subject cannot be compressed within that length then the question is useless and can under no circumstances be an aid in the elucidation of truth, consequently it should be rejected."

A very interesting paper on the subject of the medical expert witness from the facile pen of Dr. A. N. Ellis, of Maysville, Ky., may be found in *The Lancet-Clinic* of July 14, 1906.

THE BUSINESS END OF IT.

THE following irreverent expression is from the *Baltimore American*: "This," said the expert, as he looked fondly at the amount placed in his hands for his portion of the testimony, "is the kind of lunacy commission I favor."

